# Estimating the current mean age of mothers at the birth of their first child from household surveys

**DOI:** 10.1186/s12963-015-0058-9

**Published:** 2015-09-14

**Authors:** John Bongaarts, Ann K. Blanc

**Affiliations:** Population Council, 1 Dag Hammarskjold Plaza, New York, NY 10017 USA

## Abstract

**Background:**

Estimates of the period mean age at first birth are readily available for countries with accurate vital statistics (i.e., in much of the developed world). In contrast, in most developing countries vital statistics are lacking or incomplete and estimates of the period mean age at first birth are therefore often unavailable. The Demographic and Health Surveys (DHS) program provides a large set of demographic and health statistics for many developing countries, but not the mean age at childbearing or the mean age at first birth.

**Methods:**

We propose two different methods for the estimation of the period mean age at first birth from information collected in DHS surveys. The first method is the same as the one used in populations with accurate vital statistics and is based on a weighted average of single year of age first birth rates. The second is the singulate mean age at first birth.

**Results:**

A comparison of the two estimates obtained from the latest surveys in 62 countries shows excellent agreement in countries in which there is no evidence of a rise in childlessness. But, as expected on theoretical grounds, there is less agreement in populations that have experienced an increase in the proportion childless.

**Conclusions:**

Based on these results, we recommend the first method. The measure is relatively straightforward to calculate and, since it refers to recent births, is presumably more accurately reported than indicators based on events that occurred in the more distant past. This measure makes it possible for the first time to assess recent trends in the onset of childbearing in developing countries with multiple DHS surveys and to compare recent period estimates of the mean age at first birth among countries.

**Electronic supplementary material:**

The online version of this article (doi:10.1186/s12963-015-0058-9) contains supplementary material, which is available to authorized users.

## Background

Becoming a parent for the first time is one of life’s most important and influential events. It signals the onset of the responsibility for insuring the well-being and success of one’s offspring and of the next generation. For women, the age at which they have a first birth can have implications for schooling, labor force participation, and overall family size [1]. Early childbearing is also associated with elevated risks to the health of the mother and her child [2]. As a consequence, there is a large literature on the individual, social, and cultural determinants and consequences of this event and its timing in the life cycle [3–5]. In addition, a renewed interest in the wellbeing of adolescent girls has led to investments in programs intended to delay childbearing and increase access to family planning [6, 7]. Thus, the age at which women have a first birth is an important indicator of the success of these efforts. Finally, delayed childbearing slows population growth through increasing the length between generations and decreasing population momentum [8].

Estimates of both cohort and period mean ages at first birth are available for countries with reliable vital statistics. For example, EUROSTAT [9] and the Human Fertility Database [10] provide historical estimates for many countries in Europe and other high-income countries for single years from the 1980s to around 2010 and for a substantial number of birth cohorts. In contrast, in most developing countries vital statistics are lacking or incomplete and estimates of period and cohort mean ages at first birth are therefore often unavailable. The Demographic and Health Surveys (DHS) program – under which nationally representative household surveys are conducted in developing countries – provides many valuable statistics on demographic and health processes, but does not report on the period age at childbearing or age at first birth (mean or median). Instead, the standard reports provide the cohort median age at first birth as calculated from a birth history reported retrospectively by women of reproductive age. In principle, the DHS could also report the mean age at childbearing for cohorts of women but such means would be biased downward because of incomplete childbearing experience of all but the oldest women.

For many analytic purposes estimates of period measures are of greatest interest because, in contrast to cohort medians, they allow assessments of recent trends in the timing of the onset of childbearing for specific reference periods. The objective of this research note is to propose two different methods for the estimation of mean age at first birth from information collected in DHS and similar surveys. Both measures are unaffected by changes in the population age structure, thus allowing undistorted comparisons of the timing of the onset of childbearing between populations and over time within populations. Estimates are calculated for the most recent surveys in 62 countries.

## Methods

The equation for estimating the period mean age at first birth used widely in countries with vital statistics [10] is

1 $$ M(t)=\frac{{\displaystyle {\sum}_0^{a_{max}}}\left(a+0.5\right)b\left(a,t\right)}{{\displaystyle {\sum}_0^{a_{max}}}b\left(a,t\right)} $$

where

*M(t)* = Average age at first birth at time *t*

*b(a,t) =* the age-specific birth rate for birth order one at (single) age *a* and time *t*.

*a*_*max*_ = the highest age at which first births are observed

This period mean age at first birth is defined as the mean age at which women would bear their first child if they went through the reproductive years having the first birth rates observed in a particular period.

Numbers of births recorded in vital statistics are typically large and birth rates are available by single age and single year. As a result, annual estimates of *M(t)* can be estimated.

In contrast, in applications of this equation to DHS surveys samples of births in a single year are relatively small. To obtain more robust estimates of the mean age at first birth for a survey, we calculate *b(,a,t)* by single year of age for a period of three years before each survey. In addition, we exclude surveys with sample sizes of currently married women below 3000 to minimize sampling errors.

An alternative approach to estimating the period mean age at first birth is to rely on a method that is widely used to estimate the mean age at first marriage, called the “singulate mean age at marriage” [11, 12]. The application of this approach to estimate the mean age at first birth was first mentioned by Casterline and Trussell [13] and subsequently implemented by Afzal and Kiani [14] and Booth [15]. The equation is as follows

2 $$ {M}^{*}(t)=\frac{{\displaystyle {\sum}_0^{a_{max}}}p\left(a,t\right)-p\left({a}_{max},t\right)\ {a}_{max}}{1-p\left({a}_{max},t\right)} $$

M*(t) = Average age at first birth at time *t*

*p(a,t)* = Proportion of women that has not yet given birth at age *a* and time *t*

*p*_*max*_ = The proportion of women that has never had a birth at *a*_*max*_

This mean age at first birth is defined as the mean age at which women would bear their first child if they went through the reproductive years experiencing the age-specific proportions childless observed at time *t*.

In Additional file 1 we demonstrate that the two means are equal (i.e., *M* (*t*) = M* (*t*)) under the condition that the shape of the function *p(a,t)* by age is invariant with respect to time. This implies that *p(a,t)* can shift to high or lower ages over time (with corresponding changes in first birth rates and in the mean age) but with no change in shape and with constant *p*_*max*_.

Estimates of *M(t)* and M*(t) were obtained with equations (1) and (2) for the most recent DHS surveys in 62 developing countries for which data files are available for public use (and with sample sizes of married women above 3000).^1^ The number of respondents in each survey varies but typically is between 5000 and 10,000 women of reproductive age. For many countries several surveys are available, so time series of *M(t)* and M*(t) can also be calculated. Further details about the surveys are available on the DHS website [16].

Estimates of *b(a,t)* are obtained from birth histories with a simple variant of the standard DHS method for calculating age-specific birth rates by age for the three years before the survey [17]. To estimate *b(a,t)* two changes are made in this method: (1) birth rates are calculated by single year rather than by five year age intervals and (2) the numerators of the birth rates exclude births of order two and higher. Estimates of *p(a,t)* are also calculated with a variant of the standard DHS method estimating the proportion nulliparous by single year of age rather than five year age intervals.

Finally, it should be noted that values of *p(a,t)* are subject to substantial sampling errors at ages above 40, because the proportions childless at these ages are usually less than five percent and the number of respondents is smaller than at lower ages. To minimize the effects of these errors on estimates of the mean age at firth birth, the value of *a*_*max*_ is set at 40 years and *p*_*max*_ is estimated as the average of single age values of *p(a,t)* between ages 35 and 45*.*

## Results

**Fig. 1:**
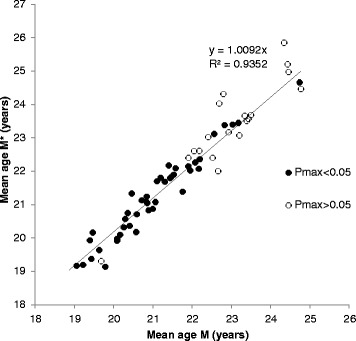
Period mean age at first birth (M* vs M)

Figure 1 plots the estimates of *M(t)* on the horizontal axis and the value of M*(t) on the vertical axis. Each marker represents the most recent survey in each of the 62 countries. The results are presented in two clusters: the solid markers represent surveys in which *p*_*max*_ is less than 5 % and the open circles represent surveys with *p*_*max*_ >5 %. This distinction is made to separate observations in which the conditions are met for *M(t)* to be equal to M*(t) from observations in which they are not. As noted in Additional file 1, a key condition for the equality of *M(t)* and M*(t) is that *p*_*max*_ is constant. Unfortunately, it is not easy to determine the rate of change in *p*_*max*_, because some countries have only one survey and, even in countries with multiple surveys, the rate of change in *p*_*max*_ is erratic due to small sample sizes. Instead, we assume that countries with *p*_*max*_ less than 5 % have seen little change in *p*_*max*_ over time, thus approximating the condition that *p*_*max*_ is constant. In surveys where *p*_*max*_ is higher than 5 % there has likely been change over time because early in the fertility transition *p*_*max*_ is typically a very small number.

It is therefore expected that *M(t)* is closer to M*(t) for surveys in the cluster with *p*_*max*_ <5 %. As is evident from Fig. 1, this is indeed the case. For these surveys the average value of *M(t)* and M*(t) are respectively 21.0 and 21.2, a difference of only 0.2 year (which is not statistically significant). However, the agreement is not perfect and the solid markers are spread around the diagonal in Fig. 1 with a standard error of 0.32 years.^2^ The second cluster of countries with open circles includes several surveys in which M*(t) is usually substantially higher than *M(t)*. This finding is likely attributable to an upward bias in M*(t) when values of *p*_*max*_ are rising (the rare cases in this cluster with *M(t)* higher than M*(t) are probably attributable to measurement or reporting errors). Our working assumption therefore is that *M(t)* is an unbiased estimator of the mean age at first birth even in surveys in the second cluster. In addition, all except one of the countries in the second cluster have a mean age at birth of 22 or higher. This result is not unexpected as there tends to be a positive correlation between age at first birth and the proportion of women who remain childless.

A full analysis of levels and trends in all 62 countries is beyond the scope of this methodological study, but a few findings can be noted. Estimates of *M(t)* vary widely among countries from a low of 19.1 in Niger (2006) to a high of 24.7 in the Maldives (2009). The unweighted averages of *M(t)* for countries in each of four regions are presented in Table 1. The low value for sub-Saharan Africa is unsurprising since this continent has not progressed as far through the fertility transition as the other regions. North Africa/West Asia and South Asia have the highest averages and Latin America has intermediate values.

**Table 1:** Average and standard deviation of country estimates of *M(t)* by region

	Average of *M(t)*	Standard deviation	N
Sub-Saharan Africa	20.9	1.1	33
Latin America	21.7	0.9	11
South Asia	22.7	1.6	10
North Africa/West Asia	23.3	1.1	8

**Fig. 2:**
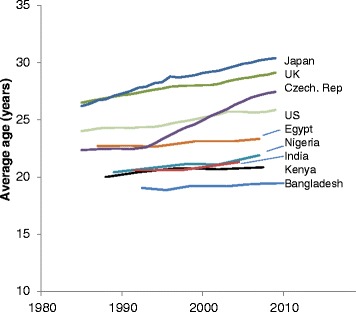
Period mean age at first birth for selected developing and developed countries

Figure 2 presents trends in *M(t)* for selected countries in the developing and developed world. Estimates for Egypt, Nigeria, India, Kenya, and Bangladesh show very modest increases from the 1990s to near 2010. The mean ages at first birth for the Japan, Czech Republic, UK, and US are mostly substantially higher and have been rising at a more rapid pace than in the five developing countries included in the figure.

As noted, DHS published reports provide estimates of the retrospectively reported cohort median age at first birth. These medians are estimated from birth histories obtained from respondents of reproductive age. The age at first birth is calculated by subtracting the woman’s date of birth from the date of birth of her first child. Medians for the cohorts aged 25–29 at the time of the survey and above are available for nearly all DHS surveys because the medians are reached before age 25 (i.e., at least half of women have had a birth before age 25). For a small number of surveys medians are available for the cohorts aged 20–24 when the median is below age 20.

These cohort medians have the advantage of being available for all DHS surveys but there are also drawbacks: 1) the median refers to past experience of cohorts and is therefore not as current as is preferable for many analytic purposes; 2) the retrospective reporting of the date of the first birth may suffer from recall errors that are likely to increase as the time since the event rises; and 3) the cohort median as calculated by DHS is not independent of the quantum of first births and can change over time even if the mean is constant. The first two of these disadvantages also apply to cohort mean ages at first birth, a measure we do not discuss because it is very rarely used as it can only be estimated accurately for women who have completed their childbearing.

**Fig. 3:**
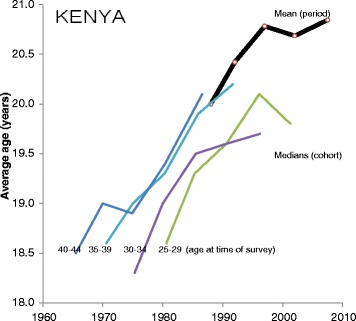
Period mean and cohort median age at first birth, based on five surveys in Kenya

To illustrate, Fig. 3 presents the estimates of the medians obtained from women aged 25–29, 30–34, 35–39 and 40–44 from six surveys in Kenya. Time series of medians are plotted as the thin lines, with one line for each of the age groups of women. Each data point is plotted in the year in which a given cohort reaches its median age. For example, if women aged 30 to 34 reported a median age at first birth of 20 years in a survey conducted during 2010 then this data point is plotted at 1998.0 years. This assumes that women aged 30 to 34 are on average 32.5 years old and with a median age at first birth of 20 years, their first birth occurred 12.5 years before the survey (i.e., age at survey – median age at first birth = 32.5–20 = 12.5). The reference date to which the median age at first birth applies is therefore 12.5 years before the survey date (i.e., reference date of survey – time before the survey to which the median age at first birth refers = 2010.5–12.5 = 1998.0). This approach allows the comparison of cohort medians reported in different surveys and of cohort and period means [18, 19]. With fully accurate reporting of the timing of first births the lines of medians plotted in Fig. 3 would exactly overlap (assuming no selectivity of migration and mortality). For example, women aged 35–39 should report a median that is the same as the median reported by women aged 25–29 in a survey conducted ten years earlier. The fact that the lines do not match indicates misreporting. In particular, it seems that the older cohorts have moved the time of the first birth closer to the survey date so that their reported medians are higher for most years than the medians reported by younger cohorts for the same years. This pattern is consistent with earlier analyses of data quality undertaken by Blanc and Rutenburg [18] and Gage [20].

Figure 3 also plots the time series of the period mean age at birth, *M(t)* as a solid line based on five surveys between 1989 and 2008/9. (The points in this line are plotted 1.5 years before the survey date to account for the fact that the mean is based on births in a three year period before the survey.) The period mean shows a rise between the 1989 and 1998 surveys but remains flat from 1998 to 2008/9.

The period means and cohort medians are not directly comparable because they are different metrics of different distributions, but by plotting the data in comparable years (as discussed above) some tentative conclusions can be reached. In particular, the medians reported by women aged 25–29 are lower than the means. This pattern is as expected because the distribution of first births is skewed to higher ages. Comparisons of period means and cohort medians in other countries yield broadly similar results (data not shown).

It should be emphasized that survey data and any measures derived from them are subject to various reporting and non-reporting errors including omission of births, displacement of births in time, and variations in sample selection and implementation [18, 21–24]. In particular, misreporting of the date of recent births has implications for assessing levels and trends in fertility. As shown by Schoumaker [24], in a number of countries with DHS surveys such errors are non-trivial and lead to underestimation of total fertility rates (TFR). Given that the calculation of *M(t)* is based on recent births, the known biases in the reporting of distant first births by older women are likely to be minimized. Interestingly, our estimate of *M(t)* remains unaffected if errors are proportionally the same at all ages. The reason is that age-specific birth rates *b(,a,t)* appear in the numerator and the denominator of Equation 1. An error of say 10 % in all *b(,a,t)* values would lead to an error of 10 % in the TFR, but there would be no error in *M(t).* In reality errors in *b(,a,t)* are likely to vary somewhat by age and that would lead to a bias in *M(t).* Furthermore, errors in birth histories would not affect M*(t), unless women misreport their childlessness status at the time of the survey.

In addition to reporting errors in the birth history, the mean age at first birth estimates could be biased by women’s misreports of their own date of birth, especially if the misreporting is linked to fertility. If, for example, a woman who has begun childbearing early overstates her age due to negative social norms around early childbearing or if an interviewer estimates her age based on her childbearing status (in places where knowledge of birth dates is uncommon), then the mean age at birth would be overestimated. The completeness and accuracy of birth date reporting, of both women and their children, is likely to have improved over time, a factor that should be kept in mind when assessing trends.

## Conclusion

The timing of the onset of parenthood is a key indicator used in studies of the determinants and consequences of early childbearing as well as an indicator of the success of various programmatic interventions. Annual estimates of the period mean age at first birth from vital statistics are widely available in most developed countries. In contrast, vital statistics of high quality are lacking in the large majority of developing countries and sample surveys such as the DHS are the primary source of demographic and health indicators. The published indicators from these data include the retrospectively reported median but not the period mean age at first birth. Both medians and means are dependent on the quality of reporting in the birth history as well as reporting of their own birth dates by women.

We assessed two methods to estimate the period mean age at first birth. The first method is the same as the one used in populations with accurate vital statistics, and the second is the singulate mean age at first birth. A comparison of the two estimates obtained from 62 DHS surveys shows excellent agreement in countries in which there is no evidence of an increase in childlessness. But, as expected on theoretical grounds, there is less agreement in populations that have experienced a rise in the proportion childless. We therefore prefer the first method. The measure is readily calculated as a straightforward variant of the standard procedure used by DHS to estimate period fertility rates and its reference period (the three years prior to the survey) is the same as the published total fertility rates. In addition, it refers to recent births and is, therefore, presumably more accurately reported than indicators based on events that occurred in the distant past. Since this new measure makes it possible for the first time to assess recent trends in the onset of childbearing in developing countries with multiple DHS surveys and to compare recent period estimates of the mean age at first birth among countries, we suggest that it be considered for inclusion in published DHS reports.

## Additional file

Additional file 1:
**Estimating the mean age at first birth.** Includes two equations that provide alternative estimates of the age-standardized mean age at first birth [25]. (DOCX 17 kb)

## Abbreviations

DHS: Demographic and health surveys
TFR: Total fertility rates

## Footnotes

1 DHS surveys do not provide estimates of birth rates or proportions ever having a birth for women under age 15. However, in the average survey 2.3 % of 15 year olds have ever given birth and very small proportions of all births therefore occur below age 15. These are estimated as follows: The proportion ever having a birth at age 14 is assumed to be one third of the proportion at age 15. The proportion ever having a birth at age 13 is assumed to be one third the proportion at age 14, etc. Age-specific birth rates under age 15 are calculated directly from these proportions.

2 Furthermore, the two means are not exactly comparable because the first method estimates the mean for the three years before the survey and the second method estimates the mean at the time of the survey. As a result, the timing of the means is about 18 months apart. This implies that when childbearing is being postponed, the first mean is slightly lower than the second. For example if the mean is rising at a rate of 1 year per decade (i.e., 0.1 per year) then the two means will differ by 0.15 years.
