# Supplementary material for: Estimating the current mean age of mothers at the birth of their first child from household surveys
Source: Popul Health Metr. 2015 Sep 14;13:25. doi: 10.1186/s12963-015-0058-9 (PMC4570715; doi:10.1186/s12963-015-0058-9)
Supplement: Additional file 1: — Estimating the mean age at first birth. Includes two equations that provide alternative estimates of the age-standardized mean age at first birth [25]. (DOCX 17 kb) [file 12963_2015_58_MOESM1_ESM.docx]

**Appendix: Estimating the mean age at first birth**

Equations (1) and (2) provide two alternative estimates of the age standardized mean age at first birth. The question to be addressed here is under which conditions these two means are equal.

We begin by making the simplifying assumption that birth rates do not change over time (i.e. *b(a,t)=b(a)* and *p(a,t)=p(a)*). Under this assumption (and in the absence of selection effects due to mortality and migration) the relationship between *b(a)* and *p(a)* is given by

$b\left( a \right)=-\frac{dp\left( a \right)}{da}$ (A1)

Substituting (A1) in (1) and changing to continuous notion yields

$M\left( t \right)=\frac{\int_{0}^{max} a\frac{dp\left( a \right)}{da}da}{\int_{0}^{max} \frac{dp\left( a \right)}{da}da}=\frac{\int_{0}^{max} adp\left( a \right)}{\int_{0}^{max} dp\left( a \right)}=\frac{-p\left( a_{max} \right).p\left( a_{max} \right)+\int_{0}^{max} p\left( a \right)d\left( a \right)}{1-p(amax)}=M^{*}$ (A2)

Where $a_{max}$ equals the highest age at which first births occur. This expression is familiar to demographers because it is widely used to estimate the so called singulate mean age at marriage (11)

Next we consider a population in which birth rates do change over time. In that case equation (A1) acquires a second term on the right side (see Bongaarts and Feeney [25] for details)

$b\left( a,t \right)=-[\frac{\delta p\left( a,t \right)}{\delta a}+\frac{\delta p\left( a,t \right)}{\delta t} ]$ (A3)

Substitution of (A3) in (1) yields
 $M\left( t \right)=\frac{\int_{0}^{max} a[\frac{\delta p\left( a,t \right)}{\delta a}+\frac{\delta p\left( a,t \right)}{\delta t} ]da}{\int_{0}^{max} [\frac{\delta p\left( a,t \right)}{\delta a}+\frac{\delta p\left( a,t \right)}{\delta t} ]da}$ (A4)

This expression cannot be simplified without adding more constraints. Bongaarts and Feeney [25] show that if the distributions *p(a,t)* shifts over time while holding its shape constant (i.e. *p(a+z,t)= p(a, t-x))* where z represents the amount of the shift between *t-x* and *t*), then

$\frac{\delta p\left( a,t \right)}{\delta t}=-r(t)\frac{\delta p\left( a,t \right)}{\delta a}$ (A5)

where *r(t)* equals the rate at which $M^{*}(t)$ is changing:

$r\left( t \right)=\frac{dM^{*}\left( t \right)}{dt}$ (A6)

Substitution of (A5) in (1) gives

$M\left( t \right)=\frac{\int_{0}^{max} a[\frac{\delta p\left( a,t \right)}{\delta a}-r(t)\frac{\delta p\left( a,t \right)}{\delta a} ]da}{\int_{0}^{max} [\frac{\delta p\left( a,t \right)}{\delta a}-r(t)\frac{\delta p\left( a,t \right)}{\delta a} ]da}=\frac{-p\left( a_{max} \right) a_{max}+\int_{0}^{max} p\left( a \right)d\left( a \right)}{1-p(amax)}=M^{*}(t)$ (A7)

In sum, $M(t)$=$M^{*}(t)$ even if the age distribution of birth rates changes, but this is only the case if the shape of the *p(a,t)* remains the same over time and $p\left( a_{max} \right)$ is constant.
